# A Ubiquitin Ligase Complex Regulates Caspase Activation During Sperm Differentiation in Drosophila

**DOI:** 10.1371/journal.pbio.0050251

**Published:** 2007-09-18

**Authors:** Eli Arama, Maya Bader, Gabrielle E Rieckhof, Hermann Steller

**Affiliations:** 1 Strang Laboratory of Cancer Research, The Rockefeller University, New York, New York, United States of America; 2 Howard Hughes Medical Institute, The Rockefeller University, New York, New York, United States of America; New York University, United States of America

## Abstract

In both insects and mammals, spermatids eliminate their bulk cytoplasm as they undergo terminal differentiation. In Drosophila, this process of dramatic cellular remodeling requires apoptotic proteins, including caspases. To gain further insight into the regulation of caspases, we screened a large collection of sterile male flies for mutants that block effector caspase activation at the onset of spermatid individualization. Here, we describe the identification and characterization of a testis-specific, Cullin-3–dependent ubiquitin ligase complex that is required for caspase activation in spermatids. Mutations in either a testis-specific isoform of Cullin-3 (Cul3_Testis_), the small RING protein Roc1b, or a Drosophila orthologue of the mammalian BTB-Kelch protein Klhl10 all reduce or eliminate effector caspase activation in spermatids. Importantly, all three genes encode proteins that can physically interact to form a ubiquitin ligase complex. Roc1b binds to the catalytic core of Cullin-3, and Klhl10 binds specifically to a unique testis-specific N-terminal Cullin-3 (TeNC) domain of Cul3_Testis_ that is required for activation of effector caspase in spermatids. Finally, the BIR domain region of the giant inhibitor of apoptosis–like protein dBruce is sufficient to bind to Klhl10, which is consistent with the idea that dBruce is a substrate for the Cullin-3-based E3-ligase complex. These findings reveal a novel role of Cullin-based ubiquitin ligases in caspase regulation.

## Author Summary

Caspases are a family of proteases that play important roles in programmed cell death (apoptosis). These enzymes also have nonlethal functions, for example, in inflammation, cell differentiation, and cellular morphogenesis. During maturation, sperm cells eliminate the majority of their cytoplasm and organelles as they are transformed into highly specialized DNA delivery vehicles. Although caspase activation does not kill the entire cell in this case, sperm maturation resembles apoptosis in the sense that many cellular structures are degraded. An important unresolved question is how the lethal activity of apoptotic caspases is regulated to prevent the unwanted death of cells. Here, we show that a Cullin-3–based enzyme complex is required for caspase activation during sperm differentiation in Drosophila. Cullins are known to target cellular proteins for degradation, but their role in caspase regulation was not previously recognized. Our results suggest that a specific Cullin-3 enzyme complex activates caspases by degrading potent caspase inhibitors, thereby providing a model for how apoptotic proteins are regulated during cellular remodeling. Importantly, components of this Cullin-3 enzyme complex are also required for fertility in mice and humans, indicating that this mechanism has been conserved in evolution from fruit flies to humans.

## Abstract 3

This work suggests that a specific Cullin-3 enzyme complex activates caspases by degrading potent caspase inhibitors.

## Introduction

Caspases are a family of cysteine proteases that have received considerable attention because of their critical roles in inflammation and apoptosis [1–4]. Caspases are expressed as weakly active zymogens in virtually all cells of higher metazoans, and their conversion to the active enzyme is tightly controlled by many different signaling pathways. Historically, most efforts to understand the mechanism of caspase regulation have focused on activator proteins, such as Apaf-1 and FADD, which promote the assembly of active initiator caspase protein complexes [5–9]. Once activated, apoptotic initiator caspases cleave and activate effector caspases, which in turn cleave a variety of important cellular targets, thereby promoting the execution of cell death [10,11]. Given the widespread expression of pro-caspases that have the potential to auto-activate in a proteolytic cascade, one might expect that efficient mechanisms exist to prevent inappropriate caspase activation in cells that should live. Furthermore, activation of apoptotic effector caspases does not always result in cell death. For example, apoptotic caspases have been shown to play a critical role for cell differentiation, proliferation, NF-κB signaling, and dendritic pruning [1,3,12–21]. At this time, the mechanisms that prevent unwanted cell killing by restricting caspase activity are poorly understood, but there are strong reasons to explore the role of inhibitory proteins. One important family of caspase inhibitors are the inhibitor of apoptosis proteins (IAPs), which can bind to and inhibit active caspases in both insects and mammals [22,23]. The most compelling evidence for a critical role of IAPs in caspase regulation has come from studies in Drosophila. Drosophila IAP1 (Diap1) encodes an E3 ubiquitin ligase that is strictly required to prevent inappropriate caspase activation and apoptosis [24–27]. In live cells, Diap1 promotes the ubiquitination and degradation of the apoptotic initiator caspase Dronc, and mutations in the RING domain of Diap1 that abrogate E3-ligase activity lead to a dramatic increase of Dronc protein, effector caspase activation, and cell death [28,29]. On the other hand, in cells that are destined to undergo apoptosis, Diap1 is inactivated by Reaper-family (RHG) proteins [24,26,27]. Reaper stimulates the self-conjugation and degradation of Diap1, thereby irreversibly removing this critical caspase inhibitor [30]. Likewise, induction of apoptosis in thymocytes induces the auto-ubiquitination and degradation of mammalian IAPs [31]. These and other observations reveal a critical role of the ubiquitin pathway in the regulation of apoptosis [30,32–37]. Ubiquitin-mediated protein degradation is a tightly regulated process, in which proteins are tagged with ubiquitin moieties through a series of enzymatic reactions involving an E1-activating enzyme, E2-conjugating enzyme, and E3 ubiquitin ligase, which determines substrate specificity. Tagged proteins are then degraded by the 26S proteasome [38–40]. However, thus far no other E3 ligases besides IAPs have been implicated in the direct regulation of caspases.

Here we provide evidence that a Cullin-3–based multiprotein complex plays a critical role in caspase activation in Drosophila. Cullins are major components of another type of E3 ubiquitin ligase that serve as scaffolds for two functional modules: a catalytic module, composed of a small RING domain protein that recruits the ubiquitin-conjugating enzyme, and a substrate recognition module that binds to the substrate and brings it within proximity to the catalytic module [41,42]. The human genome encodes seven different Cullins: Cullin-1, −2, −3, −4A, −4B, −5, and −7 [41,42]. The SCF (Skp1-Cullin-1-F-box) complexes are, so far, the best-characterized Cullin-dependent E3 ligases. More recently, the molecular composition and function of the Cullin-3–dependent E3 ligase complex has also been described [43–48]. In this complex, Broad-complex, Tramtrack and Bric-a-Brac (BTB) domain-containing proteins mediate binding of the Cullin to the substrate, whereas the Skp1/F-box heterodimer fulfill this function in the SCF complex [41,42,49]. During the past decade Cullins have been implicated in a variety of cellular activities [41]. However, very little is known about their involvement in the regulation of caspase activation and apoptosis. Here, we describe the identification of *cullin-3* mutants from a genetic screen for mutants that abrogate effector caspase activation during terminal differentiation of Drosophila spermatids. In this process, also known as spermatid individualization, spermatids eliminate the majority of their cytoplasm and organelles in an apoptosis-like process that requires canonical cell death proteins, including apoptotic caspases [12,50]. Although caspase activation in this system does not lead to death of the entire cell, sperm individualization resembles apoptosis in the sense that many cellular structures are removed into the “waste bag,” which resembles an apoptotic corpse without the nucleus. Another example where apoptotic proteins are used for cellular remodeling is the caspase-dependent pruning of neurites [14,51]. Like in spermatid individualization, the apoptotic machinery is used in a spatially restricted way to destroy only parts of a cell [14,51–54]. In our screen, we isolated several *cullin-3* alleles with mutations in a testis-specific N-terminal Cullin-3 (TeNC) domain. We show that the small RING domain protein, Roc1b, interacts with Cullin-3 in spermatids to promote effector caspase activation. We also identified a BTB-domain protein, Klhl10, that selectively binds to the testis-specific form of Cullin-3, but not to somatic Cullin-3. Mutant alleles of *klhl10* were isolated that block effector caspase activation and cause male sterility. Finally, the giant IAP-like protein dBruce binds to Klhl10 in S2 cells, suggesting that dBruce may be a substrate for the Cullin-3–dependent ubiquitin ligase complex. Together, these results define a novel Cullin-3–dependent E3 ubiquitin ligase complex that regulates effector caspase activation in Drosophila spermatids. Given the conserved nature of these proteins, our findings may have important implications for caspase regulation in other systems.

## Results

### Mutants Defective in Effector Caspase Activation during Spermatid Individualization

During sperm development in Drosophila, a group of 64 post-meiotic spermatids remain initially interconnected by cytoplasmic bridges that result from incomplete cytokinesis [55]. These spermatids are later separated from each other by the caudal movement of an actin-based individualization complex (IC) in a process termed “individualization.” During spermatid individualization, the majority of the cytoplasm and cellular organelles are removed and get deposited into “waste bags” [55–58]. This process shares several features with apoptosis and requires apoptotic effector caspases [12,15,50,59]. To gain insight into the regulation of caspases in this system, we screened for mutants that lack staining for an antibody detecting processed caspase-3 (CM1) [15,50,60–62]. We screened a collection of more than 1,000 male-sterile mutant lines defective in spermatid individualization that were previously identified among 12,326 viable mutants [63,64]. Testes from each line were stained with CM1, and 33 CM1-negative alleles representing 22 different complementation groups were identified. However, the vast majority of male-sterile mutants were CM1-positive, even though many displayed severe defects in spermatid individualization. Therefore, consistent with our earlier observations, caspase activation at the onset of spermatid individualization is independent of many other aspects of sperm differentiation [12,50].

**Figure 1:**
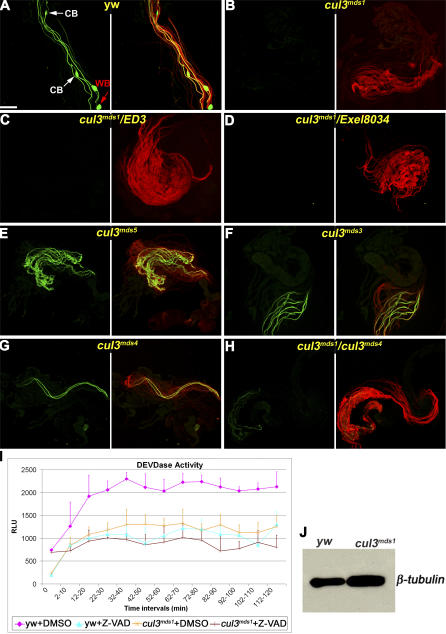
Mutations in *cul3_Testis_* Block Caspase Activation and Spermatid Individualization, but Not Axonemal Tubulin Polyglycylation (A–H) Visualization of active drICE with anti-cleaved caspase-3 antibody (CM1; green) and axonemal tubulin polyglycylation with anti-glycylated tubulin monoclonal antibody (AXO 49; red). These figures are composed of a green layer only in the left panel, and green and red layers combined in the right panel. (A) Wild-type individualizing spermatids stain positively for active effector caspase and polyglycylated axonemal tubulin (white arrows pointing at cystic bulges [CBs] and red arrow pointing at a waste bag [WB]). Elongated spermatids from (B) homozygotes for the null *cul3^mds1^* allele or (C and D) transheterozygotes for *cul3^mds1^* and two different deficiencies that cover the *cullin-3* gene, *DF*(2L)ED3 and *DF*(2L)Exel8034, respectively, stain for polyglycylation but not for active effector caspase. (E–G) Homozygote mutants for three hypomorphic *cul3_Testis_* alleles, *cul3^mds5^, cul3^mds3^,* and *cul3^mds4^,* respectively, have spermatid individualization defects but still display some levels of active effector caspase expression. (H) However, the level of active effector caspase expression was dramatically reduced in spermatids from transheterozygote mutants for the null *cul3^mds1^* and either of the hypomorphic alleles, such as *cul3^mds4^*. All the figures are in the same magnification; scale bar 200 μm. (I) The diagram depicts a DEVDase activity assay for *cul3^mds1^* ^−/−^ testes. Caspase-3–like (DEVDase) activity is detected in wild-type testes and is blocked either after treatment with the caspase-3 inhibitor Z-VAD.fmk or in *cul3^mds1^* ^−/−^ testes. DEVDase activity, presented as relative luminescence units (RLUs), was determined on Ac-DEVD-pNA substrate in testis extracts made of 180 wild-type (*yw*) or *cul3^mds1^* ^−/−^ testes treated with Z-VAD or left untreated (DMSO). Readings were obtained every 2 min, and each time interval represents an average (mean ± SEM) of five readings. Note that the level of DEVDase activity in *cul3^mds1^* ^−/−^ testes is highly similar to the corresponding level in wild-type testes that were treated with Z-VAD. (J) A Western blot analysis for the assessment of the relative protein amounts used in (I). A portion of the testis extracts in (I) were used as controls to determine the relative amounts of total protein in each extract using the anti-*β*-Tubulin antibody.

To distinguish mutants that specifically affect caspase-3 activation from ones that affect general aspects of spermatid differentiation, we used a monoclonal antibody that detects polyglycylated axonemal tubulin (AXO 49) as an advanced differentiation marker [65,66]. In wild-type spermatids, the pattern of AXO 49 staining is identical to that of CM1 (Figure S1). While mutants in eight of our complementation groups abrogated AXO 49 staining, mutations in the remaining 14 groups retained AXO 49 staining, indicating that these genes act downstream of the general signal(s) required for the initiation of spermatid individualization. One of these complementation groups was represented by five different alleles that we termed “*medusa*” (*mds*; in Greek mythology, Medusa represents both life and death). *mds1* is AXO 49–positive but completely negative for CM1 as a homozygote or in *trans* to deficiencies that cover the corresponding region (Figure 1B–1D and Figure S2). The remaining four *mds* alleles retained various levels of CM1 staining but failed to complement the sterility of *mds1,* suggesting that they are hypomorphic alleles. All these mutations were later mapped to the *Drosophila cullin-3* gene and were thus designed *cul3^mds1–5^* (Zuker lines *Z2–1089, Z2–4870, Z2–4061, Z2–1270,* and *Z2–1062*, respectively; Figure 1E–1G). *cul3^mds2^* contains an unrelated lethal mutation in the background and therefore was analyzed in *trans* to the other alleles or deficiencies in the region.

Drosophila apoptotic effector caspases, such as drICE and Dcp-1, can display DEVD cleaving activity [67–69]. We have previously shown that wild-type adult testes also contain DEVDase activity and that this activity is reduced in *cyt-c-d* mutant testes [50]. To provide independent evidence for a requirement of Cullin-3 in caspase activation, we measured DEVDase activity in *cul3^mds1^* mutant testes. Whereas lysates of wild-type testes displayed significant levels of DEVDase activity, activity in *cul3^mds1^* mutant testes was reduced to background levels, comparable to the reduction achieved with the potent caspase inhibitor Z-VAD.fmk (Figure 1I and 1J). These results confirm that *cullin-3* is required for the activation of effector caspases in spermatids.

### Genetic and Molecular Characterizations of the *cullin-3* Locus

**Figure 2:**
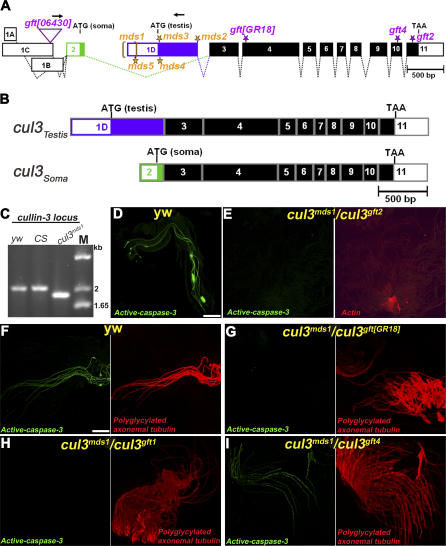
The *cul3^mds1–5^* Alleles Contain Mutations in a New Exon of the *cullin-3* Gene (A) Genomic organization of the *cullin-3* locus. Thick bars indicate exons and dotted lines indicate introns. Solid bars indicate coding sequences, whereas open bars indicate UTRs. The *Drosophila cullin-3* gene contains 14 exons, nine of which encode the bulk of the protein (exons 3–11) and are shared by both the somatic and testis-specific isoforms. While the three somatic isoforms *(cul3_Soma_)* differ in their 5′ UTRs, each beginning with a unique first exon (exons 1A, 1B, and 1C), they share a second, somatic-only exon that contains a start codon (exon 2, green bar). The testis isoform, *cul3_Testis_,* begins with a unique first exon (1D, blue bar) that includes both 5' UTR and coding sequences, including a start codon. The relative locations of the molecular lesions in *cul3_Testis_* (orange, *cul3^mds1–5^* alleles) and *cul3_Soma_* (purple, *cul3^gft2, 4, GR18^* alleles) are shown with stars (see the main text for more details on the precise molecular lesions of the *cul3^mds1–5^* alleles). The molecular alterations of the *cul3^gft^* alleles were reported in [71]: *cul3^gft06430^* contains a *PZ* element insertion 228 nucleotides from exon 2 (the insertion is indicated by a purple triangle). *cul3^gft[GR18]^* is missing a single nucleotide causing a premature stop codon at amino acid 167. *cul3^gft4^* bears a C-to-T transversion, which results in an A710-to-V conversion. *cul3^gft2^* contains a five-nucleotide deletion that results in a premature stop codon at amino acid 748 that removes half of the C-terminal Cullin homology domain (CHD). (B) A scheme of the two major mRNA isoforms of *cullin-3, cul3_Testis,_* and *cul3_Soma_*. (C) Genomic PCR and sequencing analyses of the *cullin-3* locus revealed a 181-bp deletion in *cul3^mds1^* (the arrows in A depict the relative locations of the primers used in this gPCR; *yw* and *Canton S* strains were used as wild-type controls). (D) For positive control, wild-type spermatids were stained for cleaved caspase-3 expression (green). (E) Consistent with the idea that both the *mds* and *gft* alleles affect the same gene, *cullin-3, cul3^mds1^*/*cul3^gft2^* transheterozygote mutant spermatids displayed defects in individualization and negatively stained for cleaved caspase-3 (left panel). Spermatids were counter-stained with phalloidin that binds to F-actin in the spermatids' tail (right panel, red; the strong red staining at the bottom corresponds to remnants of the testis sheath). (F–I) Spermatids were stained for cleaved caspase-3 (green in left panels) and for axonemal tubulin polyglycylation (red in right panels). (F) Wild-type control testis positively stained for cleaved caspase-3 and axonemal tubulin polyglycylation. (G–I) Whereas transheterozygous combinations between *cul3^mds1^* and the strong *cul3^gft[GR18]^* (G) or *cul3^gft1^* (H) alleles displayed spermatid individualization defects and stained negatively for cleaved caspase-3 and positively for polyglycylation, mutant spermatids from *cul3^mds1^* in trans to the hypomorphic *cul3^gft4^* allele also exhibited individualization defects but displayed reduced level of cleaved caspase-3 expression (I). All the figures were taken at the same magnification; scale bars, 200 μm.

To map the *mds* alleles, we first searched for genomic deletions that failed to complement the sterility of the *mds* males. Utilizing the “deficiency kit” from FlyBase, the male-sterility was mapped to genomic segment 35C1-35D1 on the left arm of the second chromosome (Figure S2). We then performed similar complementation tests with available mutants in this region and found that lethal *cullin-3* mutants [70,71] failed to complement the sterility of *mds* mutant males, suggesting that the *mds* alleles may represent a unique class of mutations in the *cullin-3* gene (see below, Table S1). Because the *Drosophila cullin-3* gene was previously termed *gft* [71], we will henceforth refer to the lethal *cullin-3* alleles as *cul3^gft^*. To determine the molecular nature of the *mds* mutations, we analyzed first the *cullin-3* genomic organization. The *cullin-3* gene consists of 14 exons, 11 of which contain coding sequences (Figure 2A; a partial genomic map was provided in [71]). Our genetic and molecular analyses identified a new exon, 1D, and suggested that the *cullin-3* gene codes for two major isoforms that are somatic and testis specific (Figure 2A and 2B). A lethal *P*-element insertion in the 5′ untranslated region (UTR) of the *cullin-3* gene, *cul3^gft[06430]^,* which failed to complement the lethality of other *gft* alleles, complemented the sterility of the *mds* alleles, suggesting that these noncoding sequences are only required for the somatic function of *cullin-3* (Figure 2A, Table S1, and unpublished data). Additionally, genomic PCR followed by sequencing analysis revealed that the *mds1* mutant contains a deletion in the intron that is flanked by exon 2 and exon 3, suggesting that this intron contains sequences that are only required for the function of *cullin-3* in spermatids (Figure 2A and 2C). Finally, reverse-transcriptase (RT)-PCR as well as sequence analyses of several independent clones from adult testis and somatic cDNA libraries confirmed the presence of two major *cullin-3* mRNA isoforms, *cul3_Soma_* and *cul3_Testis_* (Figure 2A and 2B; see Materials and Methods for details about the cDNA clones). While both isoforms share extensive similarity (exons 3–11), *cul3_Soma_* contains a unique, 20-amino-acid-long N-terminal polypeptide (encoded by exon 2), and *cul3_Testis_* contains a unique, 181-amino-acid-long TeNC domain encoded by exon 1D (Figure 2A and 2B; part of exon 1D is incorrectly annotated in FlyBase as an independent gene, CG31829). We also identified three different mRNA isoforms of *cul3_Soma_,* but these only differ in their 5′ UTRs (encoded by exons 1A, 1B, and 1C, Figure 2A). Cullins were previously thought to be universally expressed. Therefore, to the best of our knowledge, *cul3_Testis_* represents the first tissue-specific Cullin identified in any organism.

To determine the molecular nature of the *mds* alleles, we sequenced PCR-amplified genomic fragments of the *cullin-3* locus from these mutants. As expected from the genetic analysis, all *mds* alleles contained mutations in or near exon 1D and hence affect only the testis-specific isoform: *cul3^mds1^* has a 181-bp deletion that eliminates part of 5′ UTR of *cul3_Testis_* (orange brackets in Figure 2A and 2C). The two hypomorphic alleles, *cul3^mds3^* and *cul3^mds4^*, contain a C-to-T transversion at positions 341 and 347, which convert glutamine to stop codons at amino acids 8 and 10, respectively. As a result, translation may initiate downstream of the normal translation initiation site (orange stars in Figure 2A and Figure S3). *cul3^mds2^* contains a G-to-A transversion of a splice donor site in the intron that is flanked by exons 1D and 3; this presumably abrogates splicing between these exons (Figure 2A). *cul3^mds5^* has a G114-to-A transversion within the 5′ UTR of *cul3_Testis_* (Figure 2A). In contrast, three of the lethal *gft* alleles that failed to complement the sterility of *mds^−/−^* males—*cul3^gft[GR18]^*, *cul3^gft4^*, and *cul3^gft2^*—contain mutations in exons 4, 10, and 11, respectively, that are shared by both isoforms of *cullin-3* (purple stars in Figure 1A; [71]).

Transheterozygous combinations between *cul3^mds1^* and four strong *gft* alleles—*cul3^gft2^*, *cul3^gft[GR18]^*, *cul3^gft1^*, and *cul3^gft[d577]^*—were sterile, and their elongated spermatids were CM1-negative but AXO 49–positive (Figure 2E, 2G, 2H, Table S1, and unpublished data). Other *mds/gft* combinations with weaker alleles produced reduced levels of cleaved caspase-3 staining, wild-type levels of axonemal tubulin polyglycylation, and decreased fertility (Figures 1H, 2I, Table S1, Figure S4, and unpublished data). Collectively, these results suggest that a testis-specific isoform of *cullin-3* is required for effector caspase activation and spermatid individualization.

### Expression of *cul3_Testis_* Is Restricted to the Male Germline

**Figure 3:**
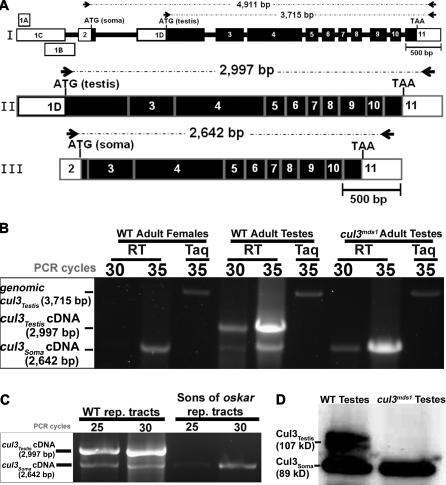
The Expression of *cul3_Testis_* Is Restricted to Male Germ Cells (A) Schematic structures of the *Drosophila cullin-3* gene (I) and of *cul3_Testis_* (II) and *cul3_Soma_* (III) mRNAs. Exons and introns are indicated by thick and thin bars, respectively. Thick black bars indicate coding sequences, whereas open bars indicate UTRs. The locations of the primers used in the comparative RT–PCR experiments in (B and C) are indicated by arrows, and the expected length sizes of the amplified fragments are indicated above each scheme. (B) Analysis of *cul3_Testis_* versus *cul3_Soma_* expression in the testis and the soma. The above primers (arrows in A) to amplify either a 2,997-bp *cul3_Testis_* or a 2,642-bp *cul3_Soma_* cDNA fragment were added to one reaction master-mix. The reaction was stopped at different cycle points to identify the linear amplification phase (30 and 35 cycles are indicated). The “RT” columns represent reverse transcriptase followed by PCR reactions, and the “Taq” are the control, PCR-only, reactions. Note that the *cul3_Testis_* expression levels were much higher in the wild-type (WT) testes than these of *cul3_Soma_*. On the other hand, only *cul3_Soma_* transcripts were detected in somatic tissues, which are represented by adult female flies. In addition, no *cul3_Testis_* transcripts were detected in *cul3^mds1^* mutant testes, confirming that this is a null *cul3_Testis_* allele. (C) The expression of *cul3_Testis_* is restricted to the male germ cells. While the expression of *cul3_Soma_* was not affected in sons of *oskar* agametic testes, no *cul3_Testis_* expression was detected. (D) Consistent with the RT-PCR analysis, no Cul3_Testis_ protein was detected in *cul3^mds1^* mutant testes on Western blot. Note, however, that expression of the Cul3_Soma_ protein in *cul3^mds1^* mutant testes was unaffected.

Our genetic analyses suggested the existence of two functionally distinct isoforms of *cullin-3, cul3_Testis_,* and *cul3_Soma_*. One possible explanation for this is that the two isoforms are differentially expressed. To test this idea, we examined the distribution of *cullin-3* transcripts in the testis and the soma. Comparative RT-PCR experiments were performed using specific primers in the unique 5′ UTRs of *cul3_Soma_* and *cul3_Testis_* and a reverse primer in their common 3′ UTR (black arrows in Figure 3A). *cul3_Soma_* was the only isoform detectable in the soma of adult females (which lack testes), and *cul3_Testis_* was the major isoform in testes (Figure 3B). Dissected testes contain both germ cells and somatic cells, such as the testicular wall, muscles cells, and cyst cells. To determine whether *cul3_Testis_* is germ-cell specific, we also analyzed RNA from a mutant lacking germ cells. Both the somatic and testis forms of *cullin-3* were expressed in wild type, but only *cul3_Soma_* was detected in the germ-cell–less reproductive tracts of adult males derived from *oskar^−/−^* mothers (Figure 3C). Since *cul3_Testis_* is not detectable in adult females, this indicates that *cul3_Testis_* expression is restricted to male germ cells, and that *cul3_Soma_* expression is mainly, if not exclusively, restricted to somatic cells (Figure 3B and 3C). Finally, consistent with the idea that promoter and 5' UTR sequences of *cul3_Testi_*_s_ are absent in *cul3^mds1^* mutants (Figure 2A), neither *cul3_Testis_* transcripts nor protein were detected in *cul3^mds1−/−^* testes. Therefore, *cul3^mds1^* has both the genetic and molecular properties of a *cul3_Testis_* null allele (Figure 3B and 3D). These results suggest that differential expression of *cul3_Testis_* in the male germline and *cul3_Soma_* in somatic tissues accounts for the distinct phenotypes (male sterility versus lethality) of the different classes of *cullin-3* mutations.

### The TeNC Domain in Cul3_Testis_ Is Required for Caspase Activation and Male Fertility

**Figure 4:**
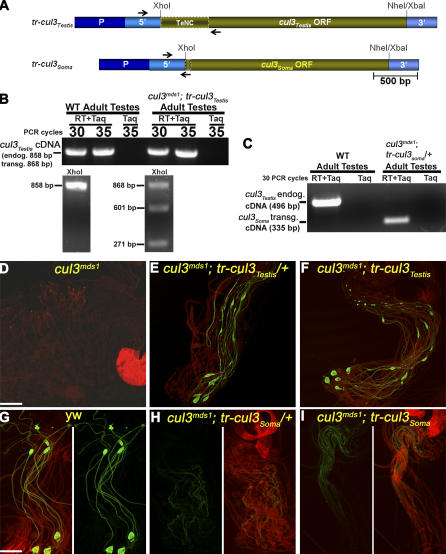
*cul3_Testis_* but Not *cul3_Soma_* Can Restore Caspase Activation and Spermatid Individualization to *cul3_Testis_* Null Mutants (A) Schematic structure of the rescue constructs for *cul3_Testis_* ^−/−^ male sterile flies. The constructs *tr-cul3_Testis_* and *tr-cul3_Soma_* are composed of the *cul3_Testis_* isoform's promoter region (dark blue, consists of the intronic sequences flanked by exons 2 and 1D) and 5′ UTR (light blue) that were fused upstream of the coding regions (ORFs) of either *cul3_Testis_* or *cul3_Soma_* followed by the 3′ UTR of *cul3_Testis_*. (B and C) Transcriptional expression from the transgenes was confirmed by RT–PCR analyses on RNA from testes of the indicated genotypes. The relative locations of the primers are indicated with black arrows in (A). “RT+Taq” and “Taq” indicate reactions with reverse transcriptase or without it, respectively, to control for possible genomic DNA contamination. (B) To differentiate between the *cul3_Testis_* endogenous (endog.) and transgenic (transg.) cDNAs, we cleaved the RT-PCR fragments with XhoI, a unique restriction site in the transgene. Note that the RT-PCR product from *cul3^mds1^*; *tr-cul3_Testis_* but not from WT testes was cleaved by XhoI, confirming its transgenic source. (C) Transgenic expression of *cul3_Soma_* (*tr*-*cul3_Soma_*) in adult testis. Note the absence of the endogenous *cul3_Testis_* cDNA band and in contrast, the presence of the transgenic *cul3_Soma_* band in *cul3^mds1^*; *tr*-*cul3_Soma_*/+ testes. (D–I) Testes stained for cleaved caspase-3 (CM1, green) and spermatid's tail and ICs (phalloidin, red). (D) Mutant spermatids for *cul3_Testis_* (*cul3^mds1^* ^−/−^) manifest a block in caspase activation and spermatid individualization. (E) Either one or (F) two copies of transgenic *cul3_Testis_* (*tr-cul3_Testis_*) restores caspase activation, spermatid individualization, and fertility of *cul3^mds1^* ^−/−^ male flies. (G) Wild-type control testes. Note the CBs and WBs (green oval structures). (H–I) In contrast, dramatically reduced CM1-positive cysts are found in *cul3^mds1^* mutants, which ectopically express (H) one or (I) two copies of the *cul3_Soma_* transgene (*tr-cul3_Soma_*). These spermatids failed to individualize, no CBs and WBs are detected and the males are sterile. Scale bars 200 μm.

The N-terminal region of Cullins is thought to mediate binding to a specific substrate recognition module [41,42] (Figure 9). To test whether the unique TeNC domain is required for the function of Cul3_Testis_, we tested whether expression of Cul3_Soma_, which lacks a TeNC domain, was able to functionally substitute for the loss of Cul3_Testis_ in developing spermatids. For this purpose, we generated transgenic flies that express the coding regions of either *cul3_Testis_* or *cul3_Soma_* under the control of the *cul3_Testis_* promoter and 5' and 3' UTRs (Figure 4A; see also Materials and Methods). At least three independent transgenic lines for each of these constructs were crossed to *cul3^mds1^* flies, and proper expression of the transgenes was confirmed by RT-PCR analysis (Figures 4B and 4C). We examined the ability of these transgenes to rescue caspase activation, spermatid individualization and male sterility of *cul3^mds1^* flies. As expected, transgenes with either one or two copies of the *cul3_Testis_* open reading frame (ORF) fully rescued CM1-staining, spermatid individualization, and male fertility (Figure 4E and 4F; note the reappearance of cystic bulges and waste bags). This proves that both the caspase and sterility phenotypes seen in *cul3^mds1^* mutant flies are due to the loss of *cullin-3* function. We next tested the ability of *cul3_Soma_* to functionally substitute for the loss of *cul3_Testis_*. Neither one nor two copies of *cul3_Soma_* rescued spermatid individualization or male fertility, although we observed very low levels of CM1-staining (Figure 4H and 4I). Since the *cul3_Soma_* and *cul3_Testis_* ORF transgenes were expressed under the same promoter and at comparable levels, we conclude that the TeNC domain is necessary for efficient caspase activation and spermatid individualization.

### *roc1b* Genetically Interacts with *cul3_Testis_* To Facilitate Effector Caspase Activation

**Figure 5:**
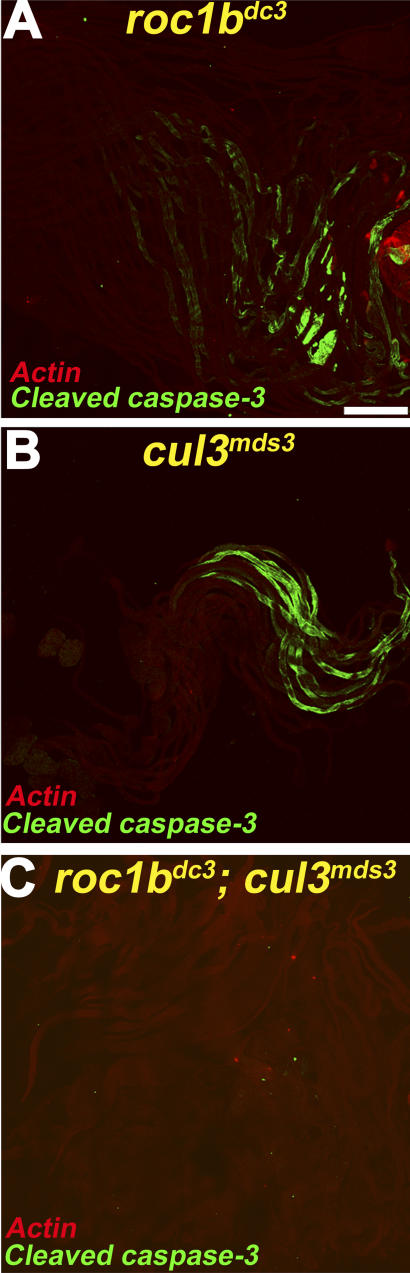
Double Mutants for *cul3_Testis_* and *roc1b* Block Caspase Activation During Spermatid Differentiation Testes stained for cleaved caspase-3 (CM1, green) and spermatid's tail (phalloidin, red). (A) Spermatids in *roc1b* mutant flies (*roc1b^dc3^* ^−/−^) display severe individualization defects and still display some levels of CM1 staining. (B) Similarly, spermatids in flies homozygous for weak *cul3_Testis_* alleles, such as *cul3^mds3^,* also display some level of CM1 staining. (C) However, spermatids mutants for both *roc1b* and *cul3_Testis_* manifest a complete block in caspase activation during individualization.

Cullins contain a C-terminal cullin homology domain (CHD) that can bind small RING domain proteins, which in turn recruit a ubiquitin-conjugating enzyme (E2) to generate the catalytic module [42,49,72]. The Drosophila genome contains three small RING domain proteins, Roc1a, Roc1b, and Roc2, all of which are capable of activating ubiquitin conjugation in vitro [73]. Loss of Roc1a function causes lethality, and targeted disruption of *roc1b* was previously reported to cause male sterility [73,74]. Furthermore, Cullin-3 preferentially co-immunoprecipitates with Roc1b, indicating that both proteins form a complex [74]. We therefore examined whether loss of *roc1b* function affects caspase activation and individualization of spermatids. We found that *roc1b^dc3−/−^* spermatids displayed reduced levels of CM1 staining and failed to individualize (Figure 5A). To test whether *roc1b* genetically interacts with *cul3_Testis_,* we generated double mutants between *roc1b^dc3^* and the hypomorphic *cul3^mds^* alleles. Homozygous mutants for either *cul3^mds^* or *roc1b^dc3^* showed moderate levels of CM1 staining (Figure 1E–1G and Figure 5B). In contrast, CM1 staining was completely abolished in spermatids of the double mutants, demonstrating that *cul3_Testis_* genetically interacts with *roc1b* to promote caspase activation in spermatids (Figure 5C). These results support the idea that Roc1b is a functionally relevant partner of Cullin-3 in vivo.

### Cul3_Testis_ Preferentially Interacts with the BTB Domain Protein Klhl10 in Yeast

**Figure 6:**
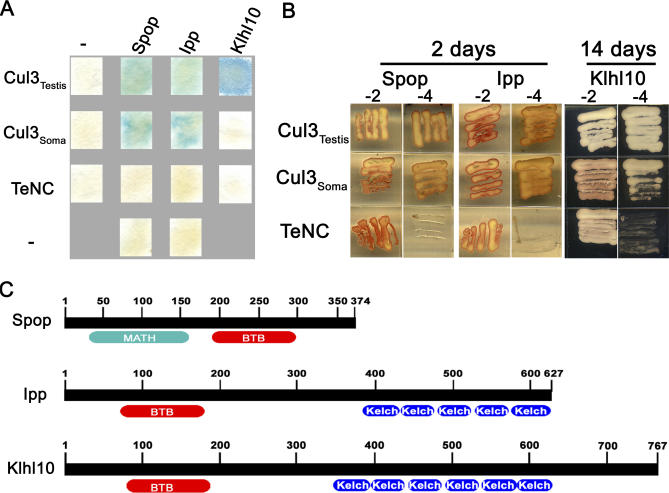
Klhl10, a BTB and Kelch Domains Protein, Preferentially Interacts with Cul3_Testis_ and Not with Cul3_Soma_ (A) Three BTB-domain proteins, the *Drosophila* orthologues of Spop, Ipp, and Klhl10, were found to interact with Cul3_Testis_ in a yeast-two-hybrid screen. β-galactosidase filter assay demonstrates that whereas Spop and Ipp can also interact with Cul3_Soma_, Klhl10 only interacts with Cul3_Testis_. While the TeNC domain of Cul3_Testis_ is required for this interaction, it is not sufficient to mediate the interaction with Klhl10. (B) Similarly, in a nutrient-omitted medium assay, yeasts with both Cul3_Testis_ and Klhl10 grew rapidly (2 d) on plates that lacked, in addition to leucine and tryptophan (−2), also histidine and adenine (−4 plates). However, yeast with Klhl10 and Cul3_Soma_ grew very poorly on −4 plates. Even after two weeks of incubation, the colony is only partially established. Note that the results for the auxotrophy rescue of Klhl10 are shown not after 2 d but rather after 14 d in order to reflect the weak interaction between Klhl10 and Cul3_Soma_. (C) Schematic representations of Spop, Ipp, and Klhl10, and the relative locations of their major domains. The BTB-domains of all these proteins are sufficient for binding to Cullin-3.

Cullin-3–dependent E3 ligases use BTB domain containing proteins for substrate recognition [44,45,49]. The number of genes encoding BTB domain containing proteins is very large, with an estimated 140–250 proteins in Drosophila [42,49]. We performed a yeast two hybrid (Y2H) screen using the coding region of Cul3_Testis_ as bait to identify potential protein partners for Cul3_Testis_ in a library of adult Drosophila cDNAs (Figure 6; see also Materials and Methods). Several cDNA clones, which encode for Drosophila orthologues of three BTB domain–containing proteins, Spop (CG9924), Ipp (CG9426), and Klhl10 (CG12423), were isolated in this screen (Figure 6). Notably, both mouse Klhl10, which shares 46% identity with its Drosophila counterpart and mouse Spop as well as Drosophila Spop were previously shown to interact with Cullin-3 [44,75–78]. Given our previous results implicating the TeNC domain in Cul3_Testis_ function, we asked whether any of these proteins bind preferentially to this domain. For this, we examined interactions between these BTB domain–containing proteins and Cul3_Testis_, Cul3_Soma_, or the TeNC domain alone in two different yeast strains. Whereas Spop and Ipp interacted with either Cul3_Testis_ or Cul3_Soma_, Klhl10 interacted with Cul3_Testis_ only (Figure 6A and 6B). However, the TeNC domain alone was not able to bind to any of the BTB domain–containing proteins in this assay. We conclude that the TeNC domain is required but not sufficient for BTB protein binding. These results identify Klhl10 as a potential partner of Cul3_Testis_ in spermatids.

### Mutations in *klhl10* Abrogate Effector Caspase Activation during Spermatid Individualization

**Figure 7:**
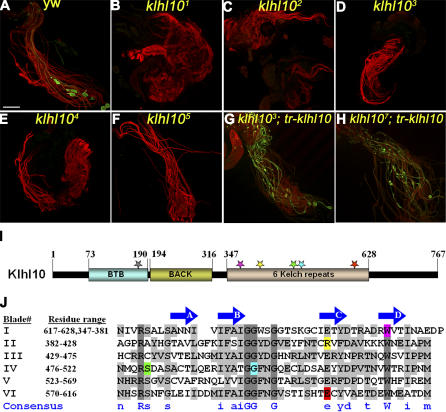
Mutations in *klhl10* Block Caspase Activation and Spermatid Individualization, but Not Axonemal Tubulin Polyglycylation (A–H) Visualization of active effector caspase with anti-cleaved caspase-3 antibody (CM1; green) and (A–F) axonemal tubulin polyglycylation with anti-glycylated tubulin monoclonal antibody (AXO 49; red) or (G–H) F-actin, which stains the ICs and the spermatids' tails (phalloidin; red). These figures are composed of combined green and red layers. (A) Wild-type individualizing spermatids positively stain for cleaved caspase-3 and polyglycylated axonemal tubulin. (B–F) In a variety of *klhl10* ^−/−^ alleles, elongated spermatids stain for polyglycylation but not for cleaved caspase-3. (G and H) Transgenic *klhl10* construct (*tr-klhl10*, composed of *cul3_Testis_* promoter and 5′ UTR, *klhl10* coding region, and *cul3_Testis_* 3′ UTR) restores caspase activation, spermatid individualization, and fertility to *klhl10* ^−/−^ male flies. (I) Schematic representation of the Klhl10 protein. The relative locations of the BTB, BACK, and Kelch domains are depicted by thick bars. Different colored stars depict the locations of the different mutations, and the colors correspond to the colored amino-acids in (J). The molecular nature of the mutations and their color code are as follows: *klhl10^2^* (*Z2–1331*) carries a G1801-to-A transversion that converts glutamic acid (E601, red) to lysine at repeat VI. *klhl10^3^* (*Z2–0960*) carries a G1119-to-A transversion that converts tryptophan (W373, purple) to stop codon, resulting in a deletion of most of the Kelch domain. *klhl10^4^* (*Z2–2739*) carries a C1237-to-T transversion that converts arginine (R413, yellow) to stop codon, which also deletes most of the Kelch repeats. *klhl10^5^* (*Z2–3284*) carries a G1486-to-A transversion that converts glycine (G496, blue) to arginine at repeat IV. *klhl10^6^* (*Z2–4385*) carries a C1439-to-T transversion that converts serine (S480, green) to phenylalanine at repeat IV. On the other hand, *klhl10^7^* (*Z2–3353*) carries a G508-to-A transversion that converts a highly conserved alanine (A170) to threonine in the BTB domain (gray star). (J) Alignment of the six Kelch repeats of Klhl10. The alignment is based on the crystal structure of the Keap1 Kelch domain which folds into a β*-*propeller structure with 6 blades. The residue range for each blade is indicated at the left. The four conserved β*-*strands in each blade are indicated above the sequences by arrows. Residues conserved in all six blades are highlighted with dark gray and appear in upper case in the consensus line, whereas residues that are conserved in at least three blades appear in lower case. Any two and above conserved residues are highlighted with light gray. Color highlighted residues are mutated in the various *klhl10^−/−^* alleles and correspond to the stars in (I).

If Klhl10 is indeed a physiologically relevant Cullin-3 binding partner in vivo, mutations in *klhl10* should affect the function of this E3 complex and thus block caspase activation. To test this hypothesis, we searched for loss-of-function mutations in this gene. Genetic analysis of the *klhl10* gene is complicated because of its position within a heterochromatic, cytologically unmapped portion on the 2nd chromosome. However, we were able to identify seven *klhl10* alleles *(klhl10^1–7^)* in our collection of CM1-defective mutants. All alleles were defective in spermatid individualization, were recessive male-sterile, failed to complement each other, lacked CM1 staining but were AXO 49–positive (Figure 7A–7F and unpublished data). This phenotype is virtually identical to the loss of Cul3_Testis_ function. By using RT-PCR and sequence analyses, we identified mutations in six of these *klhl10* alleles (Figure 7I and 7J). Five of these alleles, *klhl10^2–6^* (Zuker lines *Z2–1331, Z2–0960, Z2–2739, Z2–3284,* and *Z2–4385*) have mutations in highly conserved amino acids of the Kelch repeats, a domain that mediates interaction with the substrate (colored stars and amino acid residues in Figure 7I and 7J, respectively; more details on the molecular nature of these mutations are in the legends for Figure 7). A sixth mutation, *klhl10^7^ (Z2–3353)* contains a G508-to-A transversion that converts a highly conserved alanine (A170) to threonine in the BTB domain (gray star in Figure 7I). No mutations were identified in the ORF of *klhl10^1^ (Z2–1827),* suggesting that this allele carries a mutation in a regulatory region. To prove that these mutations are indeed responsible for the observed phenotypes, we conducted transgenic rescue experiments. Expression of the *klhl10* coding region under the control of the *cul3_Testis_* promoter (together with *cul3_Testis_* 5′ and 3′ UTRs, see Materials and Methods) completely restored CM1 staining and rescued all the sterility phenotypes associated with *klhl10* mutant alleles (Figures 7G and 7H). Collectively, these results suggest that Cul3_Testis_ interacts functionally and physically with Roc1b and Klhl10 to promote caspase activation and spermatid individualization in Drosophila.

### Elevated Level of Ubiquitinated Protein Expression in Individualizing Spermatids Requires an Intact Cul3-Roc1b-Klhl10 Complex

**Figure 8:**
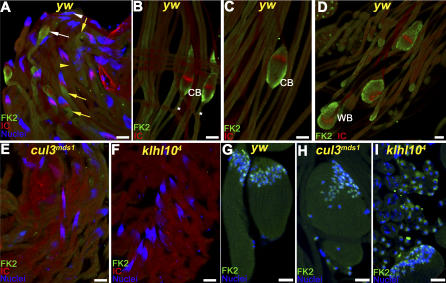
The Cul3-Roc1b-Klhl10 Complex Promotes Protein Ubiquitination during Spermatid Individualization Testes were stained with the anti–multi-ubiquitin monoclonal antibody (FK2) that detects ubiquitinated proteins (green), phalloidin, which marks the individualization complex (IC, red), and DAPI to visualize the nuclei (blue). (A) Before the formation of an IC, very low levels of ubiquitinated proteins are detected (yellow arrowhead). Once a mature IC is assembled in the vicinity of the nuclei, a steep gradient of ubiquitinated protein staining is detected from the very bottom of the nuclei to the tip of the spermatids' tails (yellow arrows). After the caudal translocation of the IC, ubiquitinated proteins are no longer detectable in the post-individualized portion of the spermatids (the region between the nuclei, indicated by a white arrowhead, and an early CB, indicated by a white arrow). (B and C) When the bulk cytoplasm of the spermatids accumulates in a cystic bulge (CB), ubiquitinated proteins are prominent within the CB and the pre-individualized region (white asterisks). (D) Once all the cytoplasm is stripped away, ubiquitinated proteins are detectable only in the waste bag (WB). (E and F) Elongated spermatids from either *cul3^mds1^* or *klhl10^4^* mutants, respectively, did not stain for ubiquitinated proteins. (G) Protein ubiquitination is detected in nuclei of early elongating spermatids. (H and I) The pattern of protein ubiquitination at earlier stages of spermatid maturation is not affected in *cul3^mds1^* and *klhl10^4^* mutants. Scale bars, 100 μm.

Our results suggest that a Cul3-Roc1b-Klhl10 E3 ubiquitin ligase complex functions at the onset of spermatid individualization. To explore this further, we investigated the level and spatiotemporal distribution of ubiquitinated proteins during spermatid individualization, and the consequences of loss of Cul3_Testis_ and Klhl10 function on this pattern. For this purpose, we stained wild-type testes with the FK2 monoclonal antibody, which specifically detects ubiquitin-conjugated proteins but not free ubiquitin. At the onset of individualization, a steep gradient of ubiquitinated protein expression is detected from the nuclear heads of the spermatids to the tips of their tails (yellow arrows in Figure 8A). During the caudal translocation of the IC, ubiquitinated proteins became completely depleted from the newly individualized portion of the spermatids (the region that is flanked by a white arrowhead and a white arrow in Figure 8A). The staining remained abundant, however, in the pre-individualized portion of the spermatids, with the highest levels seen in the cystic bulge (CB; Figure 8A–8C). At the end of individualization, the newly formed waste bag (WB) contained high levels of ubiquitinated proteins (Figure 8D). This spatiotemporal pattern of protein ubiquitination is very similar to the distribution of active effector caspase (compare Figure 8A–8D to Figure S1B and S1E or to Figure 2 in [12]). This striking correlation supports the idea that protein ubiquitination facilitates effector caspase activation in individualizing spermatids. Next, to test whether the observed ubiquitination process depends on an intact Cul3-Klhl10 complex, we stained *cul3_Testis_* and *klhl10* mutant spermatids with the FK2 antibody. The overall level of protein ubiquitination was dramatically decreased in elongated spermatids from both mutants (Figure 8E and 8F). Furthermore, this reduction was specific to late elongated spermatids, because protein ubiquitination during early stages of spermatid maturation was not significantly affected in *cul3_Testis_^−/−^* and *klhl10^−/−^* flies (Figure 8G–8I). These results show that protein ubiquitination during spermatid individualization is largely mediated by the Cul3-Klhl10 complex. Therefore, we conclude that the Cul3-Roc1b-Klhl10 complex is functionally active as an E3 ubiquitin ligase to promote protein ubiquitination during spermatid individualization.

**Figure 9:**
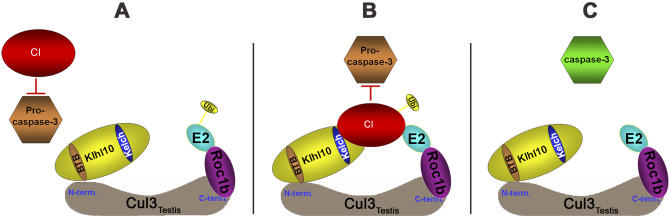
Working Model for a Cullin-3–Based Ubiquitin Ligase Complex in the Testis The diagram represents the assembly of the testis-specific Cullin-3–containing ubiquitin ligase (E3) and its proposed function in caspase activation during spermatid individualization. (A) The model suggests that once an active Cul3_Testis_-Roc1b-Klhl10 E3 ubiquitin ligase complex is assembled, it recruits a caspase inhibitor “CI” protein (red) via the Kelch domain of the substrate recruitment protein Klhl10 (yellow). A candidate for this caspase inhibitor is dBruce, a giant BIR-domain-containing ubiquitin-conjugating enzyme [12,35,36,103,104]. dBruce can physically interact via its BIR domain with Klhl10 (see Figure 10), and loss of *dbruce* function leads to spermatid death [12]. Therefore, dBruce has biochemical and genetic properties expected for the postulated “CI” protein. (B) Next, the ubiquitin-conjugating enzyme (E2, blue), which is recruited by the RING finger protein Roc1b (purple), ubiquitinates the “CI” protein. (C) Subsequent degradation of this inhibitor allows the activation of caspases at the onset of spermatid individualization process.

### dBruce, a Giant IAP-Like Protein, Can Bind the Substrate-Recognition Protein Klhl10

**Figure 10:**
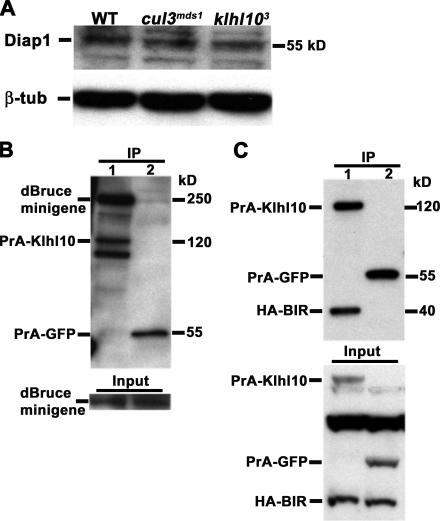
Diap1 Levels Are Not Affected in the Absence of the Functional Cul3-Roc1b-Klhl10 Complex, but dBruce Can Interact with the Substrate Recruitment Protein Klhl10 in S2 Cells (A) Diap1 protein levels were not affected in *cul3^mds1^* and *klhl10^3^* mutant testes, as assessed by Western blotting of protein extracts from dissected testes. Therefore, Diap1 does not appear to be a major target for the Cul3-based E3-ligase complex. β*-*tubulin protein levels served as loading control. (B andC) Co-IP experiment in S2 cells indicate that Klhl10 can bind to the BIR domain of dBruce. The immunoprecipitate (IP) is shown at the top, and pre-incubation of whole lysates are shown at the bottom (Input). Cell lysates were incubated with IgG beads which bind to Protein A (PrA). For Western blotting of IPs, (B) anti-dBruce antibody or (C) anti-HA antibody were used. (B) Cells were co-transfected with a *dbruce* “mini gene” (consisting of the first N-terminal 1,622 amino acids, including the BIR domain, and the last C-terminal 446 amino acids that contain the UBC domain) and (lane 1) PrA-klhl10 or (lane 2) PrA-GFP (see Materials and Methods for details). (C) Cells were co-transfected with HA-tagged dBruce-BIR peptide containing the first N-terminal 387 amino acids of dBruce that includes the BIR domain region (amino acids 251–321). This motif is sufficient to bind to Klhl10 in S2 cells (see Materials and Methods for details).

Our results suggest a simple working model in which the Cul3-Roc1b-Klhl10 complex promotes caspase activation via ubiquitination and degradation of a caspase inhibitor (Figure 9). The best-characterized family of endogenous caspase inhibitors is the IAP family [23,79]. Diap1 is essential for the survival of most, if not all somatic cells [24,27,28,30,32,80,81]. However, it appears that Diap1 is not the major caspase inhibitor in this context. If Diap1 was a substrate for Cullin-3–mediated protein degradation, we would have expected to see an increase of this protein in *cul3* mutants. However, no significant differences in Diap1 protein levels between wild-type and *cul3_Testis_^−/−^* and *klhl10^−/−^* mutant testes were detected (Figure 10A).

Another candidate is the giant, 4852–amino acid-long, IAP-like protein dBruce. dBruce function is necessary to protect sperm against unwanted caspase activity, because loss of *dbruce* function causes degeneration of spermatid nuclei and male sterility [12,24]. To further investigate possible interactions between dBruce and the Cul3-Roc1b-Klhl10 complex, we tested whether the substrate recruitment protein Klhl10 can bind to dBruce. For this purpose, we expressed tagged versions of Klhl10 and portions of dBruce in S2 cells and performed co-immunoprecipitation (co-IP) experiments (Figure 10B and 10C). In this system, Klhl10 efficiently immunoprecipitated both a dBruce “mini gene” (consisting of the first N-terminal 1,622 amino acids, including the BIR domain, and the last C-terminal 446 amino acids that contain the UBC domain; Figure 10B). Furthermore, a tagged peptide with the first N-terminal 387 amino acids of dBruce that includes the BIR domain (amino acids 251–321) is sufficient to bind to Klhl10 in this assay (Figure 10C). These data are consistent with the idea that dBruce is a substrate for the Cullin-3-based E3-ligase complex.

## Discussion

In the present study, we show a physiological requirement of a cullin-3–based E3-ubiqutin ligase complex for caspase activation and sperm differentiation in Drosophila. In this system, canonical apoptotic proteins are used to dramatically remodel cell structure by eliminating many organelles and the majority of cytoplasm [12,50]. Loss-of-function mutations in either a testis-specific isoform of cullin-3, the small RING protein Roc1b, or the BTB-Kelch protein Klhl10, all reduce or eliminate effector caspase activation in spermatids, are defective in spermatid individualization, and are male-sterile. Although mutations in many genes are known to affect male fertility, several observations indicate that the genes described here play a direct and important role in caspase regulation. First, only 3% of the male-sterile lines (33 out of 1,100) examined were negative for cleaved caspase-3 (CM1) staining, and many other mutants that block spermatid individualization and/or terminal differentiation remained caspase-positive. This confirms our earlier result that caspase activation in this system is independent of general differentiation events [12]. Secondly, mutations in any of the three *cullin-3* complex genes retain AXO 49 staining, which has a temporal pattern of expression virtually identical with CM1-staining. This shows that the Cul3-Roc1b-Klhl10 complex is required for activation of effector caspase, but not for other individualization events, such as axonemal tubulin polyglycylation. Cullin-3–based E3 ubiquitin ligase complexes were previously implicated in various biological processes, including cell-cycle control and both Hedgehog and Wnt signaling [78,82–85]. However, a role of these proteins in caspase regulation has not yet been reported. A simple working model to explain our results is that the Cul3-Roc1b-Klhl10 complex promotes caspase activation via ubiquitination and degradation of a caspase inhibitor (Figure 9). This model is further supported by the findings that the Cullin-3–based complex and effector caspases are activated in a very similar spatiotemporal pattern in individualizing spermatids, which is consistent with a direct link between effector caspase activation and protein ubiquitination in this system.

### A Testis-Specific Cullin-3 Isoform Is Exclusively Expressed in the Male Germline

We identified four different *cullin-3* transcripts, three of which are somatic and share the same coding region, and one male germ-cell–specific transcript. The latter is transcribed from a separate promoter and contains a unique first exon that encodes the N-terminal TeNC domain. This result is somewhat surprising, because there have been no previous reports of tissue-specific expression of different Cullin isoforms with distinct biochemical and functional properties. Our results indicate that the testis-specific TeNC domain plays an important role for binding to the substrate recognition protein, Klhl10. The substrate specificity of Cullin-based E3 complexes is generally achieved through a variety of substrate recognition adaptors that may be differentially available in different cell types [41,49,72]. Similar to the SCF and ECS complexes, the BTB-containing substrate recognition proteins bind via their BTB domains to the N-terminal region of Cullin-3 [49]. In the Drosophila Cul3_Testis_ isoform reported here, the TeNC domain is required for strong binding to Klhl10, caspase activation, spermatid individualization, and fertility. Cul3_Soma_, which lacks the TeNC domain but is otherwise nearly identical to Cul3_Testis_, bound much more weakly to Klhl10 and failed to rescue spermatid individualization and fertility of *cul3_Testis_* ^−/−^ flies. We conclude that the TeNC domain is important for proper binding of Cul3_Testis_ to Klhl10. However, this domain is not sufficient for binding to Klhl10, indicating that additional sequences shared between Cul3_Testis_ and Cul3_Soma_ also contribute to this interaction. The TeNC domain is highly conserved among eight different Drosophila species with an evolutionary divergence of up to 40 million years (Figure S3). Spermatozoa of D. melanogaster are 300 times longer than human spermatozoa, and other Drosophilids can produce even longer sperm, with a length up to 6 cm [86]. It is possible that the TeNC domain of Cul3_Testis_ evolved to facilitate coordinated regulation of caspase activation along the entire length of these giant spermatids. However, there is some indication that Cullin-3 can regulate caspases in tissues that do not contain a TeNC domain (see below). Therefore, it is possible that other factor(s) can substitute for the TeNC domain to promote the assembly of a similar Cullin-3–based E3 complex in species or tissues that do not express the TeNC domain.

### Role of the Ubiquitin-Proteasome System in Caspase Regulation and Cellular Remodeling

Ubiquitin pathway proteins have well-established roles in the regulation of the cell cycle, DNA damage checkpoint, signal transduction, and in the regulation of apoptosis [37,87–91]. Cullin-3–based E3 ubiquitin ligase complexes were previously implicated in various biological processes, including cell-cycle control, Hedgehog signaling, and Wnt signaling [78,82–85]. Our current study points to a previously unknown link between the ubiquitin-proteasome protein degradation system and caspase activation during late spermatogenesis. It is unlikely that the Cullin-3–based complex regulates caspases at the mRNA level, because transcripts of effector caspase *drice* and initiator caspase *dronc* are present in *cul3^mds1^* mutant testes (Figure S5). A more likely model is that the Cul3-Roc1b-Klhl10 complex promotes degradation of a caspase inhibitor (Figure 9). According to this model, the ubiquitination and degradation of this hypothetical caspase inhibitor at the onset of spermatid individualization would de-repress effector caspases and promote sperm differentiation. Whereas Diap1 is an essential caspase inhibitor in most somatic cells in *Drosophila,* it appears that it is not a major substrate for the Cul3-Roc1b-Klhl10 complex, because no significant differences in Diap1 protein levels between wild-type and *cul3_Testis_* ^−/−^ and *klhl10* ^−/−^ mutant testes were detected (Figure 10A). On the other hand, the BIR domain region of another IAP-like protein, dBruce, can bind to the substrate recruitment protein Klhl10 in S2 cells (Figure 10B and 10C). These results suggest that dBruce is at least one of the substrates for the Cullin-3–based E3-ligase complex. Importantly, it has been previously shown that loss of *dbruce* function causes degeneration of spermatid nuclei and male sterility, suggesting that dBruce function in spermatids is tightly controlled to prevent unrestrained caspase activity [12,24].

Another interesting question raised by our results is how spermatids can survive high levels of apoptotic effector caspase activity. Since transgenic ectopic expression of the effector caspase drICE leads to spermatid death (EA, MB, HS, unpublished results), we propose that caspase activity in spermatids is restricted to specific subcellular compartments. A related phenomenon has been observed during the caspase-dependent pruning of neurites [14,51]. This process is similar to spermatid individualization in that it uses the apoptotic machinery for the destruction of parts of a cell [14,51–54]. Interestingly, a requirement for the ubiquitin-proteasome system in the process of axon pruning was also reported [53]. These similarities suggest that the processes of axon pruning and spermatid individualization may use similar mechanisms to restrain the activity of apoptotic proteins for cellular remodeling. In neurons, synaptic activity can lead to local remodeling of synaptic proteins by localized proteasome-mediated degradation [92]. Likewise, it is possible that the proposed caspase inhibitor is only locally degraded, which would allow for localized caspase activity in developing spermatids.

There is some evidence that the somatic isoforms of Cullin-3 may also regulate caspase activity in other tissues. Loss of *cullin-3* function causes an increase in the number of Drosophila sensory organ precursors and external sensory organs [71]. This phenotype is reminiscent of decreased activity of the apoptotic proteins Ark, Dronc, Dcp-1, or cytochrome C-d [93–97]. Therefore, Cullin-3 may also play a role to regulate caspase activity in other non-apoptotic processes [13,81]. However, based on our results, we would expect that substrate recognition in the soma is mediated by proteins other than Klhl10.

A recent report suggest that mammalian KLHL10 and Cullin-3 can interact in vitro and that Cullin-3 is highly expressed during late murine spermatogenesis [77]. In addition, KLHL10 was shown to be exclusively expressed in the cytoplasm of developmentally advanced murine spermatids, and mice carrying a null *klhl10* allele are infertile due to defects during late spermatid maturation [98]. These data suggest that a similar E3 complex may function in late mammalian spermatogenesis and that the defects in *klhl10* mutant mice may be due to lack of caspase-3 activity. Despite apparent anatomical differences between insect and mammalian spermiogenesis, there are similarities in the removal of bulk spermatid cytoplasm. Like in insects, intracellular bridges between spermatids and the bulk of the cytoplasm are eliminated during mammalian spermatogenesis. In addition, residual bodies, which contain the extruded cytoplasm of the mammalian spermatids show high levels of active caspase-3 expression and may be homologous to the insect waste bag [99,100]. Furthermore, targeted deletion of the mouse *Sept4* locus, which encodes the pro-apoptotic protein ARTS, causes defects in the elimination of residual cytoplasm during sperm maturation [99]. Finally, a recent study reported a high frequency of mutations in *klhl10* from infertile oligozoospermic men [101]. These intriguing anatomical and molecular similarities between spermatid individualization processes in Drosophila and mammals suggest that further studies on the link between the ubiquitin-proteasome system and apoptotic proteins during sperm differentiation in Drosophila may provide new insights into the etiology of some forms of human infertilities.

## Materials and Methods

### Fly strains and expression vectors.

*yw* flies were used as wild-type controls. The Zuker mutants *Z2–1089 (cul3^mds1^), Z2–4870 (cul3^mds2^), Z2–4061 (cul3^mds3^), Z2–1270 (cul3^mds4^)*, *Z2–1062 (cul3^mds5^), Z2–1827 (klhl10^1^), Z2–1331 (klhl10^2^), Z2–0960 (klhl10^3^), Z2–2739 (klhl10^4^), Z2–3284 (klhl10^5^), Z2–4385 (klhl10^6^),* and *Z2–3353 (klhl10^7^)* were obtained from C.S. Zuker (University of California at San Diego, United States); the *gft* mutants *cul3^gft1^, cul3^gft2^, cul3^gft3^, cul3^gft4^, cul3^gftGR18^,* and *cul3^gftd577^* from M. Ashburner (University of Cambridge, United Kingdom); the *osk^[301]^*/TM3 and *osk^[CE4^* ^]/^TM3 lines from R. Lehmann (Skirball Institute, NYU School of Medicine, New York, United States); *roc1b^dc3^* from R.J. Duronio (University of North Carolina at Chapel Hill, North Carolina, United States); the deficiency lines *DF*(2L)ED3 from the Bloomington Stock Center; and the deficiency line *DF*(2L)Exel8034 from Exelixis.

The following BDGP's *cul3_Testis_* EST clones: AT08710, AT10339, AT08501, AT07783, AT21182, AT19493, and AT03216 and the *cul3_Soma_* EST clones SD20020 and RE58323 were either completely or partially sequenced, and some of them were used as templates in PCR reactions for subcloning.

The *tr-cul3_Testis_* and *tr-cul3_Soma_* rescue constructs were generated as follows: a 979-bp fragment of the presumed promoter region and 5′ UTR and a 345-bp fragment from the 3′ UTR of *cul3_Testis_* were PCR amplified from genomic DNA (forward primer CACATTGGAGCATCGTTAAA and reverse primer GAGATTGCTACGCTGGTCCA with added *Nsi*I and *Stu*I restriction sites, respectively) and the BDGP's EST clone AT07783 (forward primer GGCCCACAAAAAGTAGCA and reverse primer AGAGAATATCAAGAAATATATTAGAGGG with added *Nhe*I and *Acc*65I restriction sites, respectively), and subcloned in a sequential order into the *Pst*I + *Stu*I and *Spe*I + *Acc*65I sites, respectively, of the CaSpeR-4 vector (from V. Pirrotta). Subsequently, the complete coding regions of *cul3_Testis_* (a 2,817-bp fragment) and *cul3_Soma_* (a 2,336-bp fragment) were PCR amplified from the BDGP's EST clones AT07783 (using the forward primer ATGCAAGGCCGCGATCCCCG and reverse primer TTAGGCCAAGTAGTTGTACA with added *Xho*I and *Nhe*I restriction sites, respectively) and SD20020 (using forward primer ATGAATCTGCGGGGAAATCC and reverse primer TTAGGCCAAGTAGTTGTACA with added *Xho*I and *Nhe*I restriction sites, respectively), and ligated into the *Xho*I and *Xba*I restrictions sites between the *cul3_Testis_* 5′ and 3′ UTRs within the CaSpeR-4 vector, to generate *tr-cul3_Testis_* and *tr-cul3_Soma_,* respectively.

To generate the *tr-klhl10* rescue construct, the ORF of *klhl10* (a 2,320-bp fragment) was PCR amplified from the BDGP's EST clone AT19737 (using the forward primer ATGAGTCGTAATCAAAACG and reverse primer CTATGTACGACGACGAATTT with added *Sal*I and *Xba*I restriction sites, respectively), and ligated into the *Xho*I and *Xba*I restriction sites between the *cul3_Testis_* 5′ and 3′ UTRs within the above vector.

Standard Drosophila techniques were used to generate transgenic lines from these constructs.

### Genetic screen of the Zuker male-sterile collection lines.

The technical details of the screen were described in the supplementary 4 section in [50].

### Antibody staining.

Cleaved effector caspase antibody staining of young (0–2 d old) adult testes was carried out as described in [12] using a rabbit polyclonal anti-cleaved Caspase-3 (Asp175) antibody (CM1, Cell Signaling Technology, Cat. # 9661; http://www.cellsignal.com) diluted 1:75. The only changes are that the subsequent TRITC-phalloidin (Sigma; http://www.sigmaaldrich.com) incubation for staining of the actin filaments was carried out for 5 min in room temperature, and the slides were subsequently rinsed twice for 10 min in PBS. Axonemal tubulin polyglycylation antibody staining was carried out using the mouse monoclonal antibody AXO 49 (a kind gift from Marie-Helene Bre, University of Paris-Sud, France) diluted 1:5,000. The mouse anti-multi ubiquitin monoclonal antibody (FK2, Stressgen; http://www.assaydesigns.com) was used at a dilution of 1:100.

### Isolation of genomic DNA and sequencing of the mutant alleles.

Genomic DNA was isolated from 25–50 adult flies using the High Pure PCR Template Preparation Kit (Roche; http://www.roche.com). Genomic DNA (2 μg) was used to amplify overlapping fragments from the *cullin-3* or *klhl10* loci in wild-type and homozygote mutant lines. PCR reactions were carried out using DyNAzyme EXT DNA polymerase (Finnzymes; http://www.finnzymes.fi), according to the manufacturer instructions. The products were purified using the High Pure PCR Product Purification Kit (Roche), concentrated by evaporation, and sequenced in a GENEWIZ sequencing facility.

### DEVDase activity assay.

180 testes were dissected from newly eclosed wild-type or *cul3^mds1^* homozygote males, collected into 0.5 μl standard skirted tubes (Fisherbrand #05-669-25; http://www.fischersci.com), standing on ice and containing 70 μl of testis buffer (10 mM Tris-HCl [pH 6.8], 183 mM KCl, 47 mM NaCl, 1mM EDTA, and 1mM PMSF), homogenized using a Pellet Pestle Motor (Kontes; http://www.kimble-kontes.com/), and subsequently transferred into three new tubes (30:30:10 μl). The tubes with 10 μl of the testes extracts were used for Western blot analysis to control for the protein amount in the samples by probing with anti-*β*-tubulin antibody (E7; 1:1000; Hybridoma Bank; http://dshb.biology.uiowa.edu/). Either Z-VAD (20 μM final concentration; Enzyme Systems Products; http://www.mpbio.com/landing.php) or DMSO was added to each of the 30 μl tubes, and the samples were transferred to a 96-well assay white plate (Costar #3610, Corning; http://www.corning.com), and allowed to incubate for 10 min at RT. Caspase-Glo 3/7 reagent (Promega; http://www.promega.com) was added to a final volume of 200 μl and the signal was detected with a multiwell plate reader (SPECTRA max M2, Molecular Devices; http://www.moleculardevices.com). Luminescence readings were obtained every 2 min; therefore, each time interval in the figure represents an average of five readings. Three experiments were performed that gave similar results.

### RNA isolation and RT-PCR.

Total RNA was extracted by using the Micro-to-Midi Total RNA Purification System (Invitrogen; http://www.invitrogen.com) according to the manufacturer's recommendations. Ten–twenty young adult testes or male reproductive tracts and ten adult females were used to obtain enough RNA for 5–10 RT-PCR reactions. The samples were collected into 1.5-ml Eppendorf tubes that were standing on ice and containing 300 μl of the Invitrogen kit's lysis buffer and 3 μl of 2-mercaptoethanol, homogenized using a Pellet Pestle Motor (Kontes), and subsequently purified using the same kit. In cases when the genomic DNA had to be removed, the 30 μl of the RNA was incubated with 4 μl of RQ1 DNase and 3.8 μl of appropriate buffer (Promega; http://www.promega.com) for 1.5 h at 37 °C, and subsequently purified again with the Invitrogen kit. The RNA was stored in −80 °C or immediately used for RT-PCR reactions using the SuperScriptTM III One-Step RT–PCR System with Platinums Taq DNA polymerase (Invitrogen). The Mastercycler Gradient PCR machine (Eppendorf; http://www.eppendorf.com) was programmed as follows: 50 °C for 30 min for the RT step followed by 94 °C for 2 min, and the amplification steps of 94 °C for 30 s, 60 °C for 30 s, and 68 °C for 1 min. A master-mix was prepared and aliquoted to five tubes, each of which was amplified for 17, 20, 25, 30, or 35 cycles. Absence of genomic DNA in RNA preparations was verified by replacing the RT/Taq mix with only Taq DNA polymerase (Invitrogen). The comparative RT–PCR reactions in Figure 3 were performed using two pairs of primers in a same reaction mix: For *cul3_Testis_* the forward primer TCTCATGCAAGGCCGCGATC and the reverse primer CGGGTTATTGGCTGGCGGTC amplified a 2,997-bp cDNA fragment (and a 3,715-bp genomic fragment), whereas for *cul3_Soma_,* the forward primer CATTGATTGCCGCCGAGGAA and the reverse primer CGGGTTATTGGCTGGCGGTC amplified a 2,642-bp cDNA fragment (and a 4,911-bp genomic fragment).

For amplification of the 868-bp fragment of the transgenic *tr-cul3_Testis_* (and the 858-bp endogenous fragment) in Figure 4B, the forward primer GAGACCCGAATCGCGAGTAG and the reverse primer GCATTCTTTAAGCTGGCCCA were used. For amplification of the 335-bp fragment of the transgenic *tr-cul3_Soma_* in Figure 4C, the forward primer GAGACCCGAATCGCGAGTAG (specific for *cul3_Testis_* promoter) and the reverse primer CATTTTGCCCTCCTTCTTGG (specific for *cul3_Soma_*) were used. To simultaneously amplify a 496-bp fragment of the endogenous *cul3_Testis_,* the reverse primer GGAGGCGTTGGGCACATTGA was also used in the same reaction.

For amplification of the 538-bp fragment from *drice* mRNA in Figure S5A, the forward primer GCCCACCTTGAAGTCTCGCG and the reverse primer CAGGATGTCCAGCCGCTTGC were used. For amplification of the 527-bp fragment from *dronc* mRNA in Figure S5B, the forward primer CCACCGCCTATAACCTGCTG and the reverse primer CTGCACATACGACGAGGAGG were used.

### Plasmid construction, yeast strains, and cDNA library screening.

The Cul3_Testis_ and Cul3_Soma_ “bait” constructs were generated as follows: a 2,817-bp fragment containing the entire Cul3_Testis_ coding region was PCR amplified from the BDGP's EST clone AT19493 (forward primer CAAGGCCGCGATCCCCG and reverse primer TTAGGCCAAGTAGTTGTACA with added *Eco*RI and *Pst*I restriction sites, respectively), and a 2,336-bp fragment containing the entire Cul3_Soma_ coding region was PCR amplified from the BDGP's EST clone SD20020 (forward primer AATCTGCGGGGAAATCCTC and reverse primer TTAGGCCAAGTAGTTGTACA with added *Eco*RI and *Pst*I restriction sites, respectively). Both were subcloned in frame to the GAL4 DNA-binding domain using the *Eco*RI and *Pst*I sites of the pGBKT7 vector (Matchmaker, Clontech; http://www.clontech.com). For the TeNC domain “bait” construct, a 600-bp fragment containing the entire TeNC ORF was PCR amplified from wild-type genomic DNA (forward primer CAAGGCCGCGATCCCCG and reverse primer GGGATATTAAGACTTTCGCT with added *Eco*RI and *Bgl*II restriction sites, respectively) and subcloned in frame to the GAL4 DNA binding domain using the *Eco*RI and *Bam*HI sites of the pGBKT7 vector (Matchmaker, Clontech).

Two hybrid screens were performed using Saccharomyces cerevisiae strain AH109 and an adult Drosophila cDNA library (Matchmaker, Clontech). Selection was accomplished on synthetic complete medium lacking tryptophan, leucine, and adenine for 3–7 d at 30 °C. To test for LacZ activity, positive “prey” cDNA clones were isolated and transformed into the Y187 yeast strain, which was pre-transformed with the appropriate “bait” constructs.

The UAS-*dbruce* “mini gene” was generated as follows: a 5,022-bp fragment encoding the N-terminal 1,622 amino acids of dBruce (including the BIR domain) was cleaved by *Eco*RI and *Xho*I from the BDGP's EST clone LD31268 and subcloned into the *Eco*RI and *Xho*I sites of the pUASt vector to generate the UAS-*dbruce*-5′ vector. Next, a 1,990-bp fragment encoding the C-terminal 446 amino acids of dBruce (including the UBC domain) was cleaved with *Sal*I and *Xba*I from clone T1A-ClaI (originally identified as clone T1A in the T. Hazelrigg testis cDNA library by J. Agapite and was further cleaved with *Cla*I to remove the first 357 bp and then self ligated), and subcloned in frame into the *Xho*I and *Xba*I sites of the UAS-*dbruce*-5′ vector to generate the UAS-*dbruce* “mini gene” plasmid.

### Western blotting of adult testis, antibodies, and immunoprecipitation.

Forty to sixty testes from wild-type and mutant adult males were used to prepare extracts in 30 μl of cell lysis buffer (20 mM HEPES–KOH [pH 7.6], 150 mM NaCl, 10% glycerol, 1% Triton X-100, 2 mM EDTA, 1× protease inhibitor cocktail, and 1 mM DTT). Total protein was used for Western blot analysis using either mouse anti-CUL-3 (1:1,000; BD Transduction Laboratories, cat #611848; http://www.bdbiosciences.com) [102] or rabbit anti-Diap1 antibody [30].

To generate the anti-dBruce antibody, sequence encoding the C-terminal 446 amino acids of dBruce was cleaved from the T1A-ClaI cDNA clone (see above) using *Sal*I and *Xma*I and then cloned into the *Xho*I and *Xma*I sites of a derivative plasmid of the pET14b (Novagen; http://www.novagen.com). The expression plasmid was then transformed into BL21/DE3/pLys, followed by 2-h IPTG induction to express His_6_-dBruce-C-term. This protein was purified by nickel affinity chromatography and used to raise rat polyclonal antibody (Covance; http://www.covance.com). This antibody recognizes the dBruce “mini gene” band on a Western blot (1:1,000).

For immunoprecipitation reactions, S2 cells were co-transfected with Actin-*Gal4,* UAS-*PrA-klhl10,* and either UAS-*dbruce* “mini gene” or UAS-*HA-(dbruce)BIR* plasmids. For negative controls, S2 cells were co-transfected as above but with UAS-*PrA-GFP* instead of UAS-*PrA-klhl10*. Cells were lysed 48 h post-transfection, and the extracts were then incubated with Dynabeads which are conjugated to rabbit IgG (Dynal, Invitrogen) at 4 °C for 1.5 h. Bound proteins were eluted by boiling in 3 × SDS loading buffer and detected with anti-dBruce antibody (for the presence of dBruce “mini gene”) or anti-HA antibody (for the presence of HA-(dBruce)BIR).

## Supporting Information

Figure S1 The Spatiotemporal Expression Patterns of Axonemal Tubulin Polyglycylation and Effector Caspase Activation at the Onset of Spermatid Individualization Are Identical (A–D) Quadruple staining of wild-type spermatids with the anti-glycylated tubulin monoclonal antibody AXO 49 (red, [66]); CM1, which detects cleaved caspase-3 (green); phalloidin, which marks the individualization complex (IC, magenta); and DAPI, to visualize the nuclei (blue). (A) Red layer, (B) green layer, (C) magenta and blue layers, and (D) all 4 layers. Before or at the early assembly of an IC, neither tubulin polyglycylation nor cleaved caspase-3 is detected (yellow arrowhead). Once a mature IC is formed at the vicinity of the nuclei and is about to begin its caudal translocation, steep cascades of both tubulin polyglycylation on the axoneme and cleaved caspase-3 expression in the cytoplasm are observed from the nuclei to the tip of the tails (green arrowheads).
(A–G) When the IC moves caudally and the bulk cytoplasm of the spermatids accumulates in a cystic bulge (CB, white arrowheads), cleaved caspase-3 is removed from the post-individualized portion of the spermatids (the region between the nuclei and the CB, yellow arrows), but it is still prominent within the CB and the pre-individualized region (the region between the CB and the tip of the tail, white arrows). Cleaved caspase-3 is eventually eliminated from mature spermatozoa. On the other hand, polyglycylation of the axonemal tubulin persists during spermatid individualization and in mature spermatozoa (green arrowhead, yellow arrows and data not shown). Scale bars in (A–D), 100 μm; in (E and F), 40 μm; and in (G), 200 μm.
(10.2 MB TIF)

Figure S2 Mapping of the *mds1* Mutation The thick bar represents the cytological region between 35B1 and 35F7. The relative nucleotide positions of this region within the second chromosome are indicated above the bar. Thin bars depict available deficiencies in this region (red flanked by gray, Bloomington's deficiencies; green, DrosDel's deficiency; blue, Exelixis' deficiencies).
The *mds1* mutants were crossed to all the deficiency lines from FlyBase's second chromosome “kit” and the trans-heterozygotes were tested for male fertility. The deficiency line *Df*(2L*)TE35BC-24,* which contains a large chromosomal deletion (cytological region 35B4/6–35E1/2) failed to complement the sterility of *mds1* males, suggesting that this region covers the *mds1* mutation. Additional smaller deletion lines in this region were subsequently analyzed. The deficiencies shown in purple, *Df*(2L)ED3 and *Df*(2L)Exel8034, failed to complement, whereas the deficiencies shown in black complemented the *mds1* sterility. This genetic analysis restricted the *mds1* mutation to a 54-kb genomic interval comprising nine genes (from the *gft* gene in 35C1 to the *nht* gene in 35D1). See details in the main text on the final mapping of the *mds1* to *cullin-3*.
(524 KB TIF)

Figure S3 The TeNC Domain Has Been Highly Conserved Throughout Drosophila Phylogeny The TeNC domains of Cul3_Testis_ from eight Drosophila species were aligned using the ClustalW program (Dmel, *melanogaster;* Dsim, *simulans;* Dyak, *yakuba;* Dere, *erecta;* Dana, *ananassae;* Dpse, *pseudoobscura;* Dmoj, *mojavensis;* Dvir, *virilis*). Identical or similar amino acids are depicted by the same color. In the consensus (cons.) lines, asterisks represent residues that are identical in all the species. Square dots appear above every tenth residue, and the total numbers of amino acid in each domain appear on the right of every line in the final block. Although conservation appears throughout the domain, two regions of high conservation in the beginning and in the very end of the domain are revealed. Note that the divergence time distance between D. melanogaster and D. mojavensis or D. virilis is 40 million years.
(12.3 MB TIF)

Figure S4 The Expression Level of Cleaved Effector Caspase is *cul3_Testis_* Dose-Dependent (A–F) Cleaved caspase-3 is visualized by CM1 (green). In (C–F), the spermatids were also counter-stained with phalloidin, which detects F-actin (red). (C–F) Each figure is composed of a green layer alone (left panels) and combined green and red layers (right panels). (A) Wild-type control spermatids display cleaved caspase-3 expression. (B) Spermatids from the hypomorphic *cul3^mds^* mutants, such as *cul3^mds5^*, also display readable levels of cleaved caspase-3 expression. (C–F) However, a dramatic decrease in the level of cleaved caspase-3 expression is observed upon reduction of *cullin-3* gene copy or functionality by (C, D) crossing the hypomorphic *cul3^mds^* mutants, such as *cul3^mds2^*, *cul3^mds4^*, or *cul3^mds5^*, to deficiencies that cover the *cullin-3* locus, such as *DF*(2L)ED3, or to (E, F) the strong *cul3^−/−^* alleles, such as *cul3^gft2^*, respectively. All figures were taken at the same magnification. Scale bar, 200 μm.
(7.8 MB TIF)

Figure S5 Both *drice* and *dronc* Transcripts Are Present in *cul3^mds1^* Mutant Testes Transcriptional expressions of (A) *drice* and (B) *dronc* were confirmed by RT-PCR analyses on RNA from wild-type and *cul3^mds1^* mutant testes. “RT+Taq” and “Taq” indicate reactions with reverse transcriptase or without it, respectively, to control for possible genomic DNA contamination (see Materials and Methods for details about the primers that were used).
(181 KB TIF)

Table S1 *cul3^gft^* Lethal Mutants Failed to Complement the Sterility of *cul3^mds^* Mutant Males (28 KB DOC)

## Abbreviations

BTB: Broad-complex, Tramtrack, and Bric-a-Brac
IAP: inhibitor of apoptosis protein
IC: individualization complex
*mds*: *medusa*
ORF: open reading frame
TeNC: testis-specific N-terminal Cullin-3
UTR: untranslated region
