# Correction: A Ubiquitin Ligase Complex Regulates Caspase Activation During Sperm Differentiation in Drosophila

**DOI:** 10.1371/journal.pbio.0050291

**Published:** 2007-11-13

**Authors:** Eli Arama, Maya Bader, Gabrielle E Rieckhof, Hermann Stellar

Correction for:

Arama E, Bader M, Rieckhof GE, Steller H (2007) A Ubiquitin Ligase Complex Regulates Caspase Activation During Sperm Differentiation in Drosophila. PLoS Biol 5(10): e251. doi:10.1371/journal.pbio.0050251

In *PLoS Biology,* Volume 5, issue 10:

The academic editor's name was misspelled. It should be Erika Bach, not Erica Bach.

