# Supplementary material for: A Ubiquitin Ligase Complex Regulates Caspase Activation During Sperm Differentiation in Drosophila
Source: PLoS Biol. 2007 Sep 18;5(10):e251. doi: 10.1371/journal.pbio.0050251 (PMC1976628; doi:10.1371/journal.pbio.0050251)
Supplement: Supplementary file 1 [file pbio.0050251.st001.doc]

**Table S1. *cul3^gft^* lethal mutants failed to complement the sterility of *cul3^mds^* mutant males**

| Allelic type | ***cul3^gft1^*** | ***cul3^gft2^*** | ***cul3^gft[gr18]^*** | ***cul3^gft[d577]^*** | ***cul3^gft4^*** | ***cul3^gft3^*** | ***cul3^gft[06430]^*** |
| --- | --- | --- | --- | --- | --- | --- | --- |
| ***cul3^mds1^*** | 0 | 0 | 0 | 0 | 0 | 0 | 100 |
| ***cul3^mds2^*** | 0 | 0 | 0 | 0 | 0 | 0 | 100 |
| ***cul3^mds3^*** | 0 | 0 | 0 | 0 | 10.2±11.5 | 98.4±4.5 | 100 |
| ***cul3^mds4^*** | 0 | 0 | 0 | 0 | 0.9±0.9 | 92.5±5 | 100 |
| ***cul3^mds5^*** | 0 | 0 | 0 | 0 | 74±23 | 92.4±4.2 | 100 |

Males with transheterozygous allelic combinations of each of the indicated *cul3^gft^* alleles (upper row) in trans to each of the indicated *cul3^mds^* alleles (lefties column) were analyzed for fertility. Ten males from every transheterozygote line were allowed to mate separately with three wild-type virgin females for seven days at 25^0^C. After the parents were removed, the number of offspring was counted for 19 days. The averages and standard deviations of the total offspring numbers were calculated for each of the semi- or barley- fertile lines, and the values are presented as “percentage of fertility”, when the average fertility of wild-type males was considered as 100%. When the lines were completely sterile the value “0” was entered, whereas “100” was entered when the offspring numbers were comparable to wild-type.
